# Editorial: The role of inflammasome in neuroinflammation and neurodegeneration

**DOI:** 10.3389/fimmu.2026.1768702

**Published:** 2026-01-22

**Authors:** Suhua Chang, Yushan Guan, Qinrui Li, Pin Wan, Yongkui Li, Jinbiao Liu

**Affiliations:** 1 National “111” Center for Cellular Regulation and Molecular Pharmaceutics, Key Laboratory of Fermentation Engineering (Ministry of Education), Hubei Provincial Cooperative Innovation Center of Industrial Fermentation, School of Life and Health Sciences, Hubei University of Technology, Wuhan, Hubei, China; 2 Hubei Key Laboratory of Cognitive and Affective Disorders, Institute of Biomedical Sciences, School of Medicine, Jianghan University, Wuhan, China; 3 Laboratory of Viral Pathogenesis & Infection Prevention and Control (Ministry of Education), Institute of Medical Microbiology, Department of Immunology and Microbiology, College of Life Science and Technology, Jinan University, Guangzhou, China

**Keywords:** inflammasome, microglia, neurodegeneration, neuroinflammation, NLRP3

Neuroinflammation is now recognized as a central component of many neurological and psychiatric disorders, ranging from classical neurodegenerative diseases such as Alzheimer’s disease (AD), Parkinson’s disease (PD), Amyotrophic lateral sclerosis (ALS), and Multiple sclerosis (MS) to brain injury, vascular disease, chronic stress, and substance abuse (1, 2). As the core deriver of neuroinflammation, inflammasomes, which are multiprotein intracellular complexes that sense danger signals, promote the maturation of IL-1β and IL-18, and induce gasdermin-D-dependent pyroptosis in microglia, astrocytes, and infiltrating immune cells, thus leading to neurodegenerative diseases (3–5). Among these, the NLRP3 inflammasome has emerged as a key node linking protein aggregation, mitochondrial dysfunction, and metabolic stress to chronic inflammatory circuits in the brain (6, 7). Nevertheless, important questions remain regarding how inflammasome signaling is regulated in distinct cell types, how systemic factors such as sex and environment shape these pathways, and how they can be targeted therapeutically without compromising host defense.

**Figure 1:**
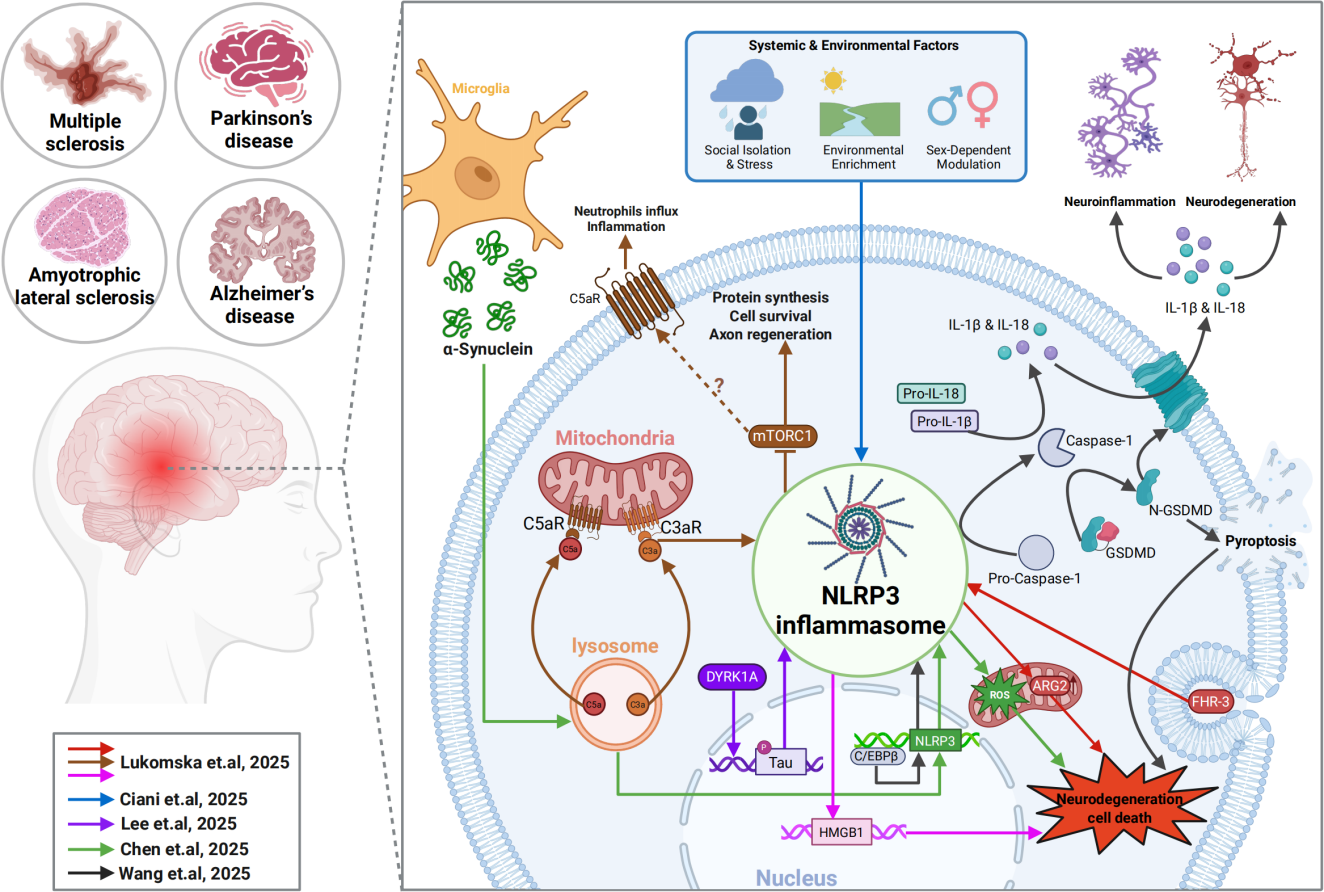
Key factors driving neurodegenerative diseases via inflammasome activation. In this Research Topic, the contributing articles focus on various factors implicated in inflammasome-associated neurodegenerative diseases. Signaling pathways from the contributed individual articles were color-indicated. Created with Biorender.com.

In this Research Topic, we bring together one original research article and four review articles that collectively explore inflammasome in the nervous system covering molecular regulation, circuit-level consequences, and systems-level perspectives. The included articles elaborate kinase-driven signaling and glial activation AD mouse models, transcriptional regulation of inflammasome components in neurodegeneration, sex- and environment-dependent modulation of NLRP3, the dual role of complement and its interplay with inflammasomes in neuronal repair, and a bibliometric analysis of microglia-associated research in PD, which highlights NLRP3 as an emerging key factor in neuroinflammation and neurodegenerative diseases (**Figure 1**). Together, these papers demonstrate how inflammasome pathways coordinate neuroimmune responses across scales—from intracellular signaling modules to broader research trends in the field.

The inflammasome contributes to AD progression by promoting the release of the proinflammatory cytokine IL-1β and reducing Aβ phagocytosis (8). Lee et al. provided complementary mechanistic and *in vivo* evidence by investigating the role of dual-specificity tyrosine phosphorylation-regulated kinase 1A (DYRK1A) in cognitive function with AD mouse models. Utilizing adeno-associated virus deliver system in wild-type, 5xFAD, and PS19 mice, they showed that hippocampal DYRK1A overexpression impaired short-term spatial/recognition memory, amplified glial activation memory, and increased neuroinflammation, whereas its knockdown improved cognitive function, reduced amyloid-beta plaques by suppressing BACE1 activity, and attenuated tau hyperphosphorylation and glial activation. Although inflammasome components are not directly quantified, the observed modulation of IL-1β expression, microglial markers, and stress-activated pathways situates DYRK1A within the broader kinase networks that converge on inflammasome-driven neuroinflammatory cascades and supports this kinase as a potential therapeutic target in AD.

The C/EBPb-inflammasome axis has emerged as a critical pathway linking transcriptional regulation to chronic inflammation in neurodegenerative diseases. Wang et al. characterized CCAAT/enhancer-binding protein beta (C/EBPβ) as a master transcriptional regulator of inflammasome signaling in neurodegenerative diseases. They summarized evidence that distinct C/EBPβ isoforms (LAP1, LAP2, and LIP) differentially modulate inflammasome activation and downstream neuroinflammation in AD, PD, ALS, and MS. In AD, C/EBPβ promoted tau cleavage, amyloid-β pathology and exacerbates neuroinflammation by upregulating APOE4, whereas in PD its silencing reduced α-synuclein aggregation and dopaminergic neuron loss in part by dampening NLRP3 activation. The authors further discussed endogenous and pharmacological modulators of the C/EBPβ–inflammasome axis, such as gut-derived metabolites and small-molecule inhibitors, while noting persistent challenges including isoform-selective targeting and blood–brain barrier penetration. By positioning C/EBPβ upstream of canonical inflammasome components, this work highlights its potential as a druggable node in neurodegenerative pathways.

Ciani et al. addressed how biological sex and environmental context shape NLRP3 inflammasome activity in the brain. The mini-review first integrated evidence and highlights sex-dependent disparities in NLRP3 signaling within systemic disorders pertinent to brain health, including CNS vascular disease, neuropathic pain, ageing, stress-related disorders, and substance use disorders. They further summarized how social isolation and environmental enrichment modulated the NLRP3 pathway to influence brain health and function, with consequences for neuroplasticity, emotional regulation, and cognitive performance. By framing NLRP3 as a sensor that integrates hormonal, genetic, and lifestyle-related inputs, the authors argued that sex and exposome should be considered primary design variables in both basic neuroimmunology and the development of NLRP3-targeted interventions.

The complement system plays vital roles in the nervous system. Lukomska et al. reviewed the complex involvement of the complement system in neurodegeneration and repair, with particular emphasis on its interaction with inflammasomes. They described how complement activation through receptors such as C3aR and C5aR1 can drive neuroinflammation, apoptosis, and pathological autophagy, partly via crosstalk with NLRP3 signaling, while in other contexts supporting synaptic pruning, neurogenesis, and axonal regeneration. A key contribution of this review is its integration of classical extracellular complement pathways with the emerging concept of the intracellular “complosome”, which links complement components to metabolic control and mTOR-dependent survival pathways within neurons. This framework underscores complement not as a simple upstream trigger of inflammation but as a bidirectional partner of inflammasomes in determining whether stressed neurons succumb or recover.

Finally, Chen et al. use bibliometric approaches to map the landscape of microglia-related research in PD. Analyzing publications from major databases with VOSviewer and CiteSpace, they identify China and the United States as leading contributors, with the Journal of Neuroinflammation as a key publication venue and Dr. Hong Jau-Shyong among the most prolific authors. Neuroinflammation, microglial activation, α-synuclein, neurodegeneration, and oxidative stress emerge as central themes, whereas gut microbiota and the NLRP3 inflammasome stand out as rapidly expanding topics. By visualizing how the field’s attention has shifted over time, this work situates inflammasome-centered microglial research within a broader network of mechanistic and translational efforts in PD and indicates where future studies are likely to concentrate.

Taken together, the contributions of this Research Topic reinforced the view that inflammasome signaling is neither an isolated cascade nor a uniform on–off switch, but a distributed regulatory system embedded in transcriptional programs, kinase networks, complement pathways, and environmental inputs. These findings highlighted the need to account for molecular regulation, sex, age, and living conditions when interpreting inflammasome-related findings and designing therapeutics strategies targeting on inflammasome in neurodegenerative diseases rather than completely abolish these pathways. Looking forward, key priorities include defining the temporal sequence and cell-type specificity of inflammasome activation across disease stages, developing reliable biomarkers of inflammasome activity in humans, and testing selective inhibitors or modulators in preclinical models that incorporate sex and environmental diversity. We expect that this Research Topic will stimulate further work on how inflammasomes coordinate neuroimmune responses in health and disease and, ultimately, contribute to the development of more precise, context-aware interventions for neurodegenerative disorders.
